# A Targeted and Tailored eHealth Weight Loss Program for Young Women: The Be Positive Be Health*e* Randomized Controlled Trial

**DOI:** 10.3390/healthcare6020039

**Published:** 2018-05-02

**Authors:** Melinda J. Hutchesson, Robin Callister, Philip J. Morgan, Ilung Pranata, Erin D. Clarke, Geoff Skinner, Lee M. Ashton, Megan C. Whatnall, Mark Jones, Christopher Oldmeadow, Clare E. Collins

**Affiliations:** 1 School of Health Sciences, Faculty of Health and Medicine, and Priority Research Centre for Physical Activity and Nutrition, University of Newcastle, Callaghan 2308, Australia; erin.clarke@uon.edu.au (E.D.C.); lee.ashton@newcastle.edu.au (L.M.A.); megan.whatnall@uon.edu.au (M.C.W.); clare.collins@newcastle.edu.au (C.E.C.); 2 School of Biomedical Sciences and Pharmacy, Faculty of Health and Medicine, and Priority Research Centre for Physical Activity and Nutrition, University of Newcastle, Callaghan 2308, Australia; robin.callister@newcastle.edu.au; 3 School of Education, Faculty of Education and Arts, and Priority Research Centre for Physical Activity and Nutrition, University of Newcastle, Callaghan 2308, Australia; philip.morgan@newcastle.edu.au; 4 School of Electrical Engineering and Computing, Faculty of Engineering and Built Environment, University of Newcastle, Callaghan 2308, Australia; Ilung.pranata@gmail.com (I.P.); geoff.skinner@newcastle.edu.au (G.S.); 5 Clinical Research Design and Statistics Support Unit, Hunter Medical Research Institute, New Lambton Heights 2305, Australia; mark.jones@hmri.org.au (M.J.); christopher.oldmeadow@hmri.org.au (C.O.)

**Keywords:** young women, weight loss, intervention, behavioural health, e-health, technology

## Abstract

Young women are gaining weight rapidly. Evidence for effective weight loss interventions targeting young women is lacking. This randomized controlled trial assessed the efficacy and acceptability of a six-month targeted and tailored eHealth weight loss program for young women (Be Positive Be Health*e* (BPBH)). Women aged 18–35 years were randomized to BPBH (*n* = 29) or control (*n* = 28). BPBH supported participants to modify diet and physical activity behaviours using evidenced-based strategies (e.g., self-monitoring) tailored for young women and delivered using e-health (website, social media, smartphone application, email, text messages). The primary outcome was a change in weight (kg) at six months. Acceptability was assessed via a process evaluation survey and usage of intervention components. No significant between-group differences were observed for weight, with significant mean differences favouring the intervention group observed for body fat (kg) (−3.10 (−5.69, 0.52), *p* = 0.019) and intakes of alcohol (g) (−0.69 (−1.33, 0.04), *p* = 0.037), vegetables (% energy/day) (4.71 (−2.20, 7.22), *p* < 0.001) and energy-dense, nutrient-poor foods (% energy/day) (−9.23 (−16.94, 1.52), *p* = 0.018). Retention, intervention usage and satisfaction were moderate. BPBH facilitated positive improvements in body fat and dietary intake, but not weight. Intervention acceptability findings support the use of some intervention components (e.g., Facebook, Smartphone app) with young women.

## 1. Introduction

Young women are gaining weight rapidly, with longitudinal studies indicating that women are gaining significantly more weight (range of 6–14 kg) during young adulthood (18–35 years) than at any other life stage [1,2,3]. This level of weight gain is of major concern given that more women now enter young adulthood already overweight or obese [4,5]. For example, in Australia, the prevalence of obesity among women aged 18–21 years increased from 8.6% to 14.6% between 1995 and 2015 [6]. Overweight and obesity in young adult women are associated with an increased risk of type 2 diabetes mellitus, cardiovascular disease (CVD), various cancers, including endometrial and breast [7,8], and all-cause mortality [9] in later life. In addition, obesity is associated with higher rates of depression, infertility and pregnancy-related adverse health, including gestational diabetes [7,8,10]. Hence, there is a need for effective interventions to optimize weight and improve health outcomes in young adult women.

Currently, the evidence is limited for the effectiveness of weight loss interventions specifically for young women, and for young adults generally. Previous systematic reviews of weight loss interventions targeting young adults have found only a small number of randomized controlled trials (RCT) reporting intervention efficacy [11,12]. These reviews of weight loss interventions targeting young adults concluded that, despite some positive improvements in weight across studies, the heterogeneity of interventions meant that the most effective interventions to achieve weight loss in young adults could not be identified. Furthermore, the authors noted trends in intervention preference by sex (i.e., males preferred exercise training, and females preferred diet interventions) [12].

Despite the proposed preference, traditional weight loss interventions incorporating diet and behavioural components still may not be effective for young women, with lower enrolment, lower engagement, e.g., session attendance, higher drop-out and less weight loss reported compared with older participants [13]. The challenges of recruiting and engaging young women in weight loss interventions are a recurrent theme in the literature [11,12,13,14]. Consequently, there is a marked need for new intervention approaches that are targeted, i.e., specifically aimed at young women, and tailored, i.e., personalized by considering individual characteristics and mediators of behaviour [15,16]. Increasingly, the evidence suggests that targeted and tailored interventions potentially lead to greater participant engagement and behaviour change [16,17]. Targeted and tailored interventions may be more engaging for young adults because the complexity of factors experienced by individuals in this transitional life stage (e.g., changes to living situation, attending university and entering the workforce) is taken into account, and therefore, they may be more effective at achieving weight loss in this at-risk group [18].

Be Positive Be Health*e* (BPBH) is a targeted and tailored e-health weight loss intervention for young women (18–35 years) and was developed based on formative research with the target group, including an online survey assessing young women’s motivations and barriers to weight change and preferences for an e-health weight loss program [19]. A beta version of the BPBH intervention was evaluated in a single-arm (*n* = 18) pre-post study, with the results at three months demonstrating significant reductions in participants’ weight and waist circumference [20]. Therefore, the primary aim of this RCT was to investigate the efficacy of the six-month BPBH program on body weight (primary outcome) and additional secondary outcomes, compared to a waiting list control group. The secondary aim was to evaluate the acceptability of the BPBH program to young women. 

## 2. Materials and Methods

### 2.1. Study Design

A six-month RCT was conducted with participants randomized to the BPBH program (intervention) or waiting list control group. Assessments were conducted at baseline and six months. Data were analysed using both intention-to-treat and complete cases. The trial was registered with the Australian New Zealand Clinical Trials Registry, Number ACTRN12615000272594. The protocol is available at https://www.anzctr.org.au/Trial/Registration/TrialReview.aspx?id=368150&isReview=true. The study protocol was approved by the University of Newcastle Human Research Ethics Committee H-2014-0138. Written informed consent was obtained from all participants.

### 2.2. Participants and Recruitment

Inclusion criteria were: female, aged 18–35 years, body mass index (BMI) 25.0–34.9 kg/m^2^, email and Internet access, iPhone model 4s or newer, social media accounts willing to be used for the study and able to attend measurement sessions. Participants were excluded if they were currently pregnant or breastfeeding, planning pregnancy in the next six months, participating in another weight loss program, taking medications that had caused weight gain, had a metabolic disorder, eating disorder or other medical condition where weight loss may compromise health, non-English speaking or had weight loss of ≥5% of initial weight in the last three months.

Participants were recruited over a one-month period in March/April 2015. They were recruited via media releases from the University of Newcastle and Hunter Medical Research Institute, posters at the university campus, local technical colleges, local businesses and organizations known to engage with the target group and the social media pages of these settings. An email invitation was also sent to consenting participants of a previous research study in young women. 

### 2.3. Intervention: Be Positive Be Healthe

Table 1 summarizes the major components of BPBH. In brief, BPBH is a six-month weight loss program delivered using e-Health technologies only, comprising five delivery modes (website, app, email, text messages and social media) and using social cognitive theory (SCT) and control theory (CT) theoretical frameworks. 

Individualized energy intake and energy expenditure goals were set for each participant based on their estimated energy expenditure (calculated using the Harris-Benedict equation including their starting weight and height) and creating a 2500 kJ/day energy deficit to help facilitate a 0.5–1 kg weight loss/week. The energy intake and energy expenditure goals were to be achieved by making changes to the eating behaviours and physical activities identified from our formative research as priorities for young women. All resources promoted the BPBH 10 key weight loss messages related to these key eating behaviours and physical activities and evidenced-based weight loss strategies (i.e., self-monitoring, stimulus control, cognitive restructuring and social support). The weight loss messages were communicated to participants as the “10 Steps to Success” as follows: 1. Boost your intake of vegetables and fruit; 2. Cut-back on eating out and take-away foods; 3. Manage your energy from drinks; 4. Move more; 5. Sit less; 6. Create an environment to help achieve your goals; 7. Control eating in response to emotions; 8. Be ready and have a positive attitude; 9. Keep track of weight, food and exercise; 10. Seek support. During the first 12 weeks of the program, a different topic was covered each week in email newsletters, text messages and social media. Weight Loss 101 and general healthy eating and physical activity guidelines were covered in Week 1, followed by each of the 10 Steps to Success consecutively (Weeks 2–11) and problem solving (Week 12). For Weeks 13–22, two of the 10 Steps to Success were re-visited each fortnight (with a focus on overcoming barriers to change and acknowledging successful behaviour change), and in Weeks 23–26, the topics were maintaining behaviour change and weight loss maintenance. Program components were further targeted to young women by considering their specific motivations for wanting to control their weight (i.e., “feel better about themselves,” “increase self-confidence,” “improve health”), as well as primary barriers to weight management (i.e., time, motivation, cost) [20]. Branding and graphic design throughout the program materials also reflected young women (e.g., images of young women, colours/fonts that appeal to women). 

BPBH participants were provided with access to program components (log-in details for website, joined Facebook group and followed Instagram account, downloaded self-monitoring app) immediately following randomization at the in-person measurement session and were instructed to follow the directions provided by the various program components once the program commenced on 27 April 2015.

### 2.4. Wait List Control Group

The wait list control group received no intervention for six months and was instructed to continue their usual eating and physical activity habits. They received access to all BPBH program components after six months. 

### 2.5. Data Collection

Assessment sessions were held at the University of Newcastle, NSW, Australia, and conducted by trained, blinded assessors at baseline and after six months. All participants fasted (minimum 8 h, maximum 12 h) prior to their assessment session. Questionnaires were completed online either prior to or during the assessment sessions. Participants who were unable to attend assessment sessions in person at six months were invited to complete the online survey and provide a self-reported weight.

### 2.6. Outcomes

#### Efficacy

The primary outcome measure was weight change (kg) from baseline to 6 months. Weight was measured to 0.01 kg in light clothing without shoes on a digital scale (Inbody 720, Inbody Australia, Miami, QLD, Australia). Height was measured to 0.1 cm using the stretch stature method on a stadiometer (Inbody BSM370; Inbody Australia, Miami, QLD, Australia). Waist circumference was measured to 0.1 cm using a non-extensible steel tape measure as the narrowest point between the lower costal border and the umbilicus. BMI was calculated using the standard equation (weight (kg)/height (m)^2^) [21]. Body fat (kg and percentage) was determined using bioelectrical impedance (Inbody 720, Inbody Australia, Miami, QLD, Australia). Blood pressure (systolic and diastolic) was measured using an automatic sphygmomanometer (Inbody BPBIO320, Inbody Australia, Miami, QLD, Australia). Participants were seated for five min prior to commencement of blood pressure measurements with a two-min rest between repeated measures. Height, weight, waist circumference and blood pressure measurements were taken twice for accuracy, with a third measurement also taken in cases where either of the first two values fell outside a predetermined acceptable range. The average of the two acceptable measurements was used in the analysis. Total, HDL and LDL cholesterol and triglycerides were measured in a blood sample obtained by finger prick and analysed using the validated Cardiochek reflectance spectrophotometer lipid measurement tool (Point of Care Diagnostics Pty Ltd., Artarmon, NSW, Australia) [22,23].

An online survey assessed physical activity, sitting time, dietary intake and quality of life. Minutes/week of moderate to vigorous activity was assessed using the Godin Leisure Time Exercise Questionnaire [24]. Total, weekday and weekend sitting time/day were assessed using the Domain-Specific Sitting Time Questionnaire [25]. Total energy intake and percentage energy/day contributed by alcohol, take-away foods, fruit, vegetables, nutrient-dense healthy foods and energy-dense, nutrient-poor (EDNP) foods, as well as total grams/day of fruit, vegetables and alcohol were assessed using the 120-item semi-quantitative Australian Eating Survey Food Frequency Questionnaire [26]. Subjective well-being was determined using the Quality of Life, Enjoyment and Satisfaction Questionnaire [27] and Satisfaction with Life Scale [28]. 

### 2.7. Acceptability

BPBH program participants completed a process evaluation survey and were asked to rank overall program acceptability/satisfaction on a 5-point Likert scale, from strongly agree (=5) to strongly disagree (=1), as well as the attractiveness (“was visually appealing”), comprehension (“provided me with useful information about”), usability (“was easy to use/receive”) and ability to persuade/engage (“helped me to attain my weight loss goals”, “motivated me” and “made me feel accountable”) of the individual program components. 

In addition, each participant’s use of intervention components was objectively tracked, including number of website log-ins, completion of online quizzes and goal setting, number of email newsletters opened, number of self-monitoring entries (weight, food or exercise) made in the Easy Diet Diary app and number of posts/likes to the Facebook group and Instagram account. Each ‘use’ was date stamped allowing usage to be tracked from Weeks 1–26 of the program. Text messages sent were objectively tracked, whereas whether text messages were read was reported in the process evaluation questionnaire (How often did you read the BPBH text messages? “Regularly”, “sometimes”, “rarely”, “never”). 

#### 2.7.1. Sample Size

For 90% power to detect a 3-kg difference in weight change between the two groups at the 5% significance level, assuming the correlation between baseline and six-month weight was 0.8 and allowing 40% loss to follow-up at six months (based on previous systematic review findings) [11], a recruitment target of 114 women (57/group) was set. 

#### 2.7.2. Randomization

The allocation sequence was generated by a computer-based random number algorithm (https://www.sealedenvelope.com/), producing individual group allocation in block lengths of six and stratified by BMI (overweight BMI: 25–29.9, obese 30–35 kg/m^2^). A researcher not involved in the study prepared concealed envelopes, which were distributed following completion of baseline assessments, by a researcher not involved in data collection.

#### 2.7.3. Statistical Analysis

Data were analysed using Stata Version 13.1 (StataCorp. 2011. Stata Statistical Software: College Station, TX, USA). Analysis for the primary and secondary outcomes was conducted on an intention-to-treat basis (all participants who were randomized to groups and completed baseline assessments) and for completers only (those who provided data at six months). The effect of treatment on the primary outcome was assessed using linear mixed models. Weight (or other secondary outcome) was the outcome in the model, time (baseline, six months) and treatment group (intervention, control) as predictors, and group × time as an interaction term. BMI was included as a co-variate in the model. The *p*-value associated with the interaction term was used to determine the statistical significance of any difference between treatment groups in the change from baseline. Effect sizes were calculated using the equation: Cohen’s *d* = (M_1 change score_ − M_2 change score_)/SD_pooled (change scores)_. Participant characteristics at baseline are presented as the mean ± SD. Program acceptability and satisfaction measures are presented as the mean ± SD, with higher scores (maximum of 5) indicating greater acceptability/satisfaction.

## 3. Results

### 3.1. Recruitment

**Figure 1:**
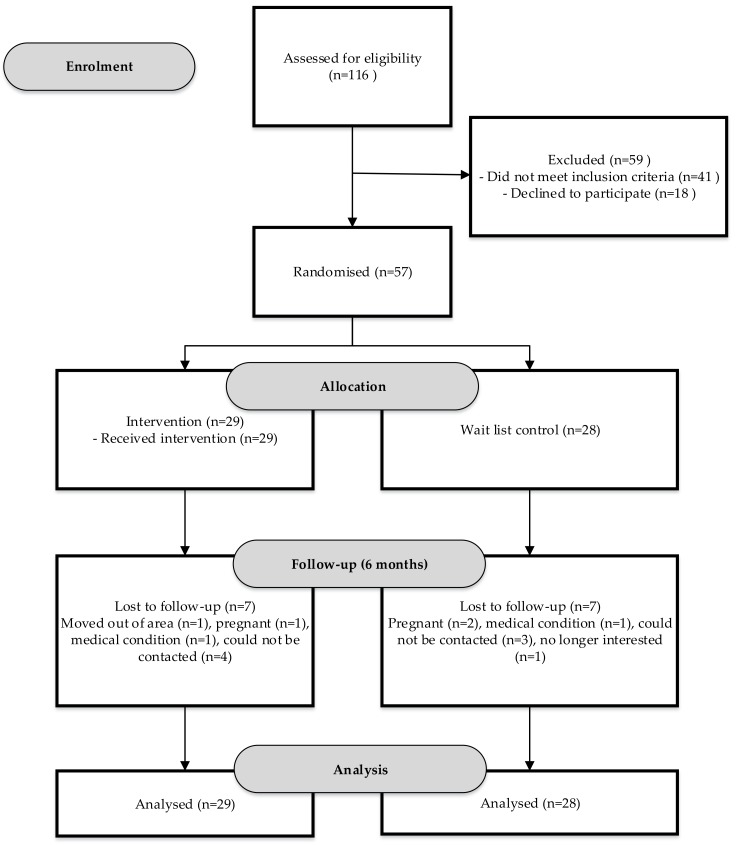
Flow diagram of young women recruited to the Be Positive Be Health*e* six-month weight loss program randomized controlled trial.

Of the 221 young women who expressed an interest in participating in the study, 116 were screened for eligibility, and 75 deemed eligible, of whom 57 were randomized to the BPBH group (*n* = 29) or control group (*n* = 28) (Figure 1). Therefore, the recruitment target of 114 participants was not met. 

### 3.2. Participants at Baseline

Participant characteristics at baseline are summarized in Table 2. Participants had a mean age of 27.1 ± 4.7 years; most (91.2%) were born in Australia; 49.1% had never been married; and 50.9% were currently studying. Individual income was diverse, with approximately one-third of the participants’ incomes classed as low, middle and high. At baseline, participants mean BMI was 29.4 ± 2.5 kg/m^2^, with 56.1% overweight and 43.9% obese.

Participants reported spending an average of 208 min/week in moderate to vigorous physical activity (~30 min/day) and 573 min/day sitting. They also reported consuming 45% of their total energy intake from EDNP foods, including 5.5% from takeaway foods. The mean Satisfaction with Life Scale score was 21.5 ± 6.5, which is within the average range (20–24) for the scale. 

### 3.3. Program Efficacy

Table 3 summarizes the results of the intention-to-treat analysis examining baseline to six-month differences between the intervention and control groups for all outcomes. No significant difference between groups in change from baseline was observed for the primary outcome of weight. Significant within-group changes in weight were found in the intervention group, regardless of whether measured weight only was included in the analysis (−2.04 kg (−4.07, −0.01), *p* = 0.049) or when both measured and self-reported weight data were included in the analysis (−1.94 kg (−3.59, −0.29), *p* = 0.022). Significant differences between groups favouring the intervention group were observed for body fat (kg, *p* = 0.019, Cohen’s *d* = −0.44), alcohol intake (g, *p* = 0.037, *d* = −0.41), vegetable intake (% energy/day, *p* < 0.001, *d* = 1.07) and intake of nutrient-dense healthy (% energy/day, *p* = 0.018, *d* = 0.77) and EDNP foods (% energy/day, *p* = 0.018, *d* = −0.76). Supplementary Table S1 provides the results of the completers’ analysis (i.e., those who provided data at six months), which were consistent with the intention-to-treat analysis, including similar effect sizes. 

Significant positive within-group changes were also found in the intervention group for BMI, body fat (kg and %), waist circumference, systolic and diastolic blood pressure, total cholesterol and daily intakes of fruit (grams), vegetables (% energy and grams), take-away foods (% energy), nutrient-dense healthy foods (% energy) and EDNP foods (% energy). Significant within-group changes were found in the control group for waist circumference.

### 3.4. Program Acceptability

The mean overall satisfaction with the program was 3.2 ± 0.9 out of a maximum of five, with 33.3% (*n* = 7) strongly agreeing/agreeing they were satisfied with BPBH and 47.6% (*n* = 10) indicating they were neutral. Mean satisfaction with the seven program components (Table 4) ranged from 3.0–3.8, with the goal setting (3.8 ± 0.7) and self-monitoring app (3.8 ± 0.8) the program components with which participants were most satisfied, whereas they were least satisfied with the website (3.0 ± 1.0). Most program components were rated as visually appealing (range: 3.0–4.0), with the self-monitoring app rated highest (4.0 ± 0.5) and the text messages lowest (3.0 ± 0.9). Comprehension for the program components was variable, ranging from 2.9–3.8. The social media posts were perceived as providing the most useful information about healthy eating, exercise and weight loss. The ability of the program components to engage/persuade participants was generally scored lower (2.4–3.8) than other survey items. The self-monitoring app scored highest for accountability, motivation and helping to attain goals, while the text messages were consistently the lowest for most acceptability measures. 

Over the six months of the intervention, 96.6% of participants (*n* = 28) logged-on to the website. The mean number of webpage visits was 52 ± 29 across the six months, ranging from 0–135. On average, participants completed 1.9 ± 1.3 of the five quizzes at Weeks 1, 3, 8, 12 and 20. In total, 21 of the 29 participants (72.4%) set goals and recorded them on the website on Week 1. Just over half (58.6%) the participants used the Easy Diet Diary application to self-monitor their food, exercise and/or weight. More specifically, 58.6% used the app to self-monitor their food intake, making an average of 164 ± 312 entries; 44.8% used it to self-monitor their exercise, making an average of 6.7 ± 11.1 entries; and 34.5% used it to self-monitor their weight, making an average of 1.1 ± 2.2 entries. Participants on average opened 10.0 ± 4.4 of the 19 email newsletters over the six months. Text messages were sent to all participants over the 26 weeks of the program, of which 52.4% reported reading them regularly. Two participants reported never receiving the text messages, and one participant reported that they only began receiving the text messages towards the end of the program. 

For Facebook, there was a total of 138 posts, 86 by the facilitator and 52 by participants. The mean number of posts by participants was 1.8 ± 2.5. There was a total of 319 ‘likes’ made on Facebook posts, with a mean of 11.0 ± 16.5 likes per participant. There were 359 comments made on Facebook, with a mean of 12.4 ± 19.8 comments per participant. For the 86 posts on Instagram, there were 54 ‘likes’ with a mean number of likes of 1.9 ± 4.7 per participant, with no comments made. 

**Figure 2:**
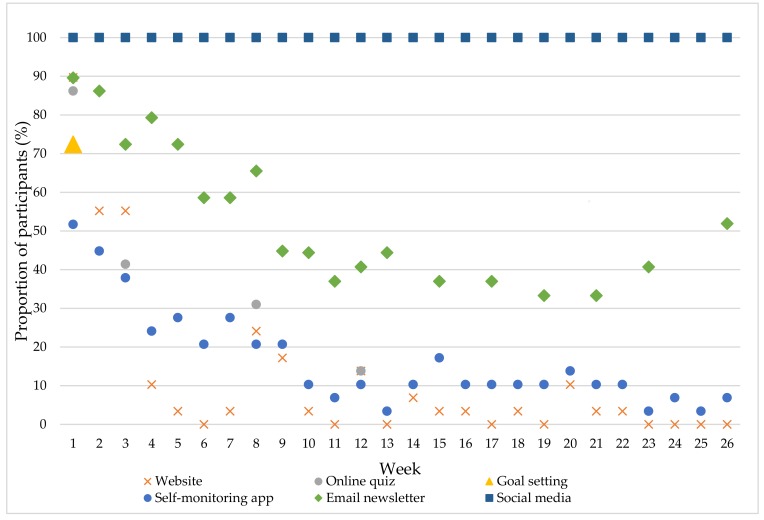
Proportion of participants who ‘engaged’ with each Be Positive Be Health*e* program component from Weeks 1–26.

Figure 2 illustrates the proportion of participants who engaged with each of the six program components from Weeks 1–26. All participants engaged with the social media accounts, particularly Facebook, throughout the six-month program. Engagement with the email newsletters was the next highest with a minimum of 33.3% of participants opening a newsletter to a maximum of 89.6%. Most participants (89.6%) accessed the website in the first week of the program, with the proportion logging-in declining thereafter. No participants logged-in to the website in Weeks 6, 11, 13, 17, 19, 23–26, and the proportion accessing during the other weeks ranged from 3.4–55.2%. Most (86.2%) participants completed the online quiz in Week 1, with 41.4% in Week 3, 31% in Week 8 and 13.7% in Weeks 12 and 20, while 72.4% of participants completed the goal setting component of the program. Just over half the participants (51.7%) accessed the self-monitoring app and self-monitored their weight, food or exercise during Week 1 of the program. Engagement with the app declined until around Week 10, where it appears a more consistent group of users was established with 10.3% of participants using the app for nine of the remaining weeks. 

## 4. Discussion

BPBH is the first targeted and tailored weight loss program designed specifically for young women and delivered entirely using e-health technologies. There were no significant between-group differences for weight compared to a waitlist control group after six months. The BPBH program did lead to significant intervention effects for secondary outcome measures, including a very large effect size for vegetable intake, large effect sizes for HDL cholesterol, percentage energy from both nutrient-dense healthy and EDNP foods and moderate effect sizes for total body fat and alcohol intake. Program retention, acceptability and engagement were modest. 

Our findings are comparable to results from the Early Adult Reduction of weight through LifestYle (EARLY) intervention trials, a consortium of seven trials developed in response to the identified need for interventions to address young (18–35 years) adults’ high prevalence of overweight and obesity [29]. Three of the trials specifically focused on weight loss: the Cell Phone Intervention for You (CITY) [30], Social Mobile Approaches to Reduce weight (SMART) [31] and Innovative approaches to Diet, Exercise and Activity (IDEA) studies [32,33]. Overall, the three trials produced only moderate initial weight loss that was not sustained in the longer term, despite substantial resource investment. More specifically, CITY tested a two-year m-health intervention with Group 1 exclusively using a smartphone application for intervention delivery and self-monitoring, while Group 2 received a six-week personal coaching intervention with follow-up phone contact and only self-monitoring using the smartphone application [30]. Only the personal coaching intervention was superior to controls in weight loss achieved (net effect −1.92 kg, *p* = 0.003), and weight loss was only significant in the short term (six months) and not at 12 or 24 months [30]. SMART compared a two-year multiple modality intervention (Facebook, mobile apps, text messaging, emails, website and technology-mediated communication with a health coach) to a control group. The difference in weight between the two groups was significantly different at six (−1.33 kg) and 12 (−1.33 kg) months, but not at 18 or 24 months [31]. Finally, IDEA involved participants receiving the same initial six months of weekly group sessions and prescribed diet and exercise, with the 470 participants then randomized to the standard (self-monitoring via website) or enhanced (monitoring via wearable activity tracker and web interface) intervention groups [32,33]. Participants in the enhanced intervention achieved less weight loss at 24 months compared to those in the standard intervention group (2.4 kg) [33]. The magnitude of weight loss in these studies is not dissimilar to the within-group changes seen in the BPBH intervention group from baseline to six months. However, a major limitation of the BPBH trial was the small sample size (*n* = 57), which meant inadequate power to detect a significant difference between groups for this moderate level of weight loss. 

Challenges associated with recruiting young adults into obesity and nutrition-related research and programs have been widely acknowledged [34,35] and this was apparent in the BPBH trial. Despite over 220 young women expressing an interest (contacting the researchers) in the study, only 116 completed the online eligibility screening survey, and only 57 of the 75 deemed eligible attended baseline assessments and were therefore randomized. Recruitment was challenged by the short duration available for recruitment (one month) due to funding deadlines. In addition, a catastrophic weather event in the Newcastle region during this timeframe, which resulted in loss of power and Internet across households in the region for up to two weeks, impacted both recruitment and baseline assessments (e.g., those who were eligible were unable to be contacted). Both the recruitment rate (26%), i.e., the proportion of individuals who expressed an interest in the study that were randomized, and participation rate (76%), i.e., the proportion of eligible participants who were randomized in this study, were low, although consistent with other obesity-related research with young adults [36]. Since recruitment to this trial in 2015, important findings in relation to recruitment of young adults to obesity-related trials have been published, including those from the EARLY trials [34,37] and a systematic review of successful recruitment strategies [38]. For example, Lam et al. reported that to recruit a sample size greater than 100, between nine and 36 months may be required [38]. Therefore, future weight loss trials with young women should continue to investigate innovative strategies to recruit young women and also ensure sufficient personnel and time are available for recruitment. 

The beneficial effects of BPBH on secondary outcomes is promising. In particular, the effects on dietary intake included significant between-group differences, with moderate to very large effect sizes for changes in intakes of vegetables, alcohol and both core and non-core foods. These findings are novel as few young adult weight loss trials have evaluated changes in dietary intake. Intervention participants increased their vegetable consumption on average by 55 g/day, which is almost three-quarters of a serving. They also decreased the proportion of energy contributed by non-core foods by 8.3%. With self-reported energy intakes of ~8500 kJ/day at baseline, this constitutes approximately a 700-kJ/day reduction in non-core food intake, which is equivalent to 1.4 servings and hence is a substantial reduction. These findings suggest that the dietary components of the intervention were successful, and this is supported by the participants’ feedback on the usefulness of information about healthy eating, which was consistently scored highly compared to other areas. Conversely, participants scored the usefulness of information about exercise and weight loss consistently lower, with no significant between-group differences found for weight, physical activity levels or sitting time. Of note, participants self-reported high levels of physical activity, with average time spent in MVPA consistent with national recommendations at baseline. These findings are not consistent with the profile of young women in Australia, with only 61% of women aged 24–35 years sufficiently active (≥150 min/week) [39]. Physical activity level was assessed by self-report and therefore may have been biased (e.g., unrealistic perception of their physical activity levels at baseline). Overall, further investigation and refinement of the intervention strategies used to improve physical activity levels and decrease sitting time in BPBH may be required to facilitate greater weight loss among young women. 

Retention in BPBH was 75% at six months, which is similar to other weight management interventions with young women [11]. A systematic review of weight management interventions for young women found only three of the eight included studies achieved retention rates of 80% or more at six months or less [11]. Similarly, Lam et al. found only 13 of 26 young adult weight gain prevention studies retained more than 80% of participants [38]. Interestingly, the recent EARLY trials appear to have overcome this pattern. Project SMART reported 94% retention at six months [31], the Choosing Healthy Options in College Environments and Settings (CHOICES) study reported 92% retention at four months [34], and the IDEA study reported 90% retention at six months [32]. The BPBH study used many similar strategies to retain participants as outlined by the EARLY trials (e.g., multiple forms of contact information, follow-up visits outside business hours). One difference was that the EARLY trials provided incentives ranging from $40–$100 at each measurement time point, whereas in BPBH participants received one AU$20 gift voucher at completion of the six month follow up. Future interventions may need to consider using similar incentives to maximize retention of young women and consequently enhance the internal study validity.

Overall, the young women were moderately engaged and satisfied with the BPBH intervention. The most positive findings were for the social media accounts, particularly Facebook, as all remained engaged throughout the trial and overall there was a high number of Facebook posts made by participants and ‘likes’ of the content posted by the facilitator. In addition, BPBH participants ranked social media as providing the most useful information for healthy eating, physical activity and weight loss, and overall it received the second highest score for satisfaction. This is consistent with the project SMART study in young adults, which reported Facebook to be the primary modality through which dynamic content was delivered at the group level [31]. Furthermore, social media is frequently used by young adults. In 2015, 90% of U.S. adults aged 18–29 years used social media [40], with Facebook being the most popular (79% of online adults reported using Facebook) [41]. Given the wide reach and relatively low cost of intervention development, further exploration of social media as a component of weight loss interventions for young adults is warranted. 

Engagement and acceptability of other program components was more variable. For most other components, there was an observable decline in engagement over time, and this is consistent with other e-health research [42,43,44,45,46]. The website and the other intervention components accessed via the website (online quizzes and goal setting) experienced the greatest decline in usage across the six months. Participants also reported being least satisfied with the website. This may have been due to the nature of the website, which was static over the six months, and therefore, multiple visits did not provide any ‘new’ content. Furthermore, participants ranked the ‘ease of use’ of the website lowest, which suggests a potential issue with access or usability of the website. Notably, the website was not responsive for smartphones and therefore may have limited some participants’ accessibility. Although the completion of the online quizzes declined over time, participants ranked these and the goal setting feature higher than the website. This suggests that the inclusion of these important behaviour change techniques (self-monitoring and goal setting) were more valued by the participants. The self-monitoring app had the lowest rate of uptake of any program component (51.7% in Week 1), but was ranked the highest for satisfaction, accountability, motivation and helping to attain goals. This acceptability may be contributed to by the commercial nature of the app and is supported by the more consistent usage patterns by the app users in this study. As self-monitoring has been consistently associated with weight loss success [44], the low uptake of the app however is disappointing and potentially contributed to the non-significant weight loss results. Further investigation of the potential reasons for this low uptake and potential strategies to motivate all participants to use this important intervention feature is warranted. 

### 4.1. Strengths and Limitations

The strengths of this study include a targeted and tailored approach with extensive intervention development for this under-represented population group. Due to recruitment challenges, the sample was small with low power and prevents any conclusions from being made regarding the generalizability of findings, so the efficacy results are indicative, rather than conclusive. Furthermore, attrition was an issue, and specific retention strategies (i.e., monetary incentives for each assessment time-point) must be in place for future trials with young women. 

### 4.2. Implications

The results of this RCT demonstrate the potential of an e-health delivered, targeted and tailored weight loss intervention for young women and also provide further evidence of the complexities associated with recruiting, engaging and retaining this population group in weight loss interventions. In terms of the BPBH intervention, future program delivery may benefit from a greater focus on social media, particularly Facebook, given its high engagement. The lower acceptability and engagement with the text messages, and to a lesser extent the email newsletters, suggests that these intervention components could be removed. Furthermore, in place of text messages and email newsletters, regular updates to the website content could be made throughout the six-month program to encourage continued engagement, which in turn may further boost engagement with the online quizzes and goal setting website features. Additional prompts or nudging could also be incorporated within the Facebook group to encourage greater engagement with the website. Finally, the self-monitoring app requires better integration within the program, particularly in the first week of the program, to facilitate greater initial uptake. For example, the ability to synchronize self-monitoring data from the app with the online quizzes and goal setting features may increase uptake and engagement. A recent review of e-health weight loss interventions acknowledged a trend for interventions delivered solely using e-health technologies to achieve less weight loss than those delivered using e-health combined with other non-e-health delivery modes (e.g., face-to-face consultations, telephone coaching) (−3.70 kg mean difference in weight loss compared to control versus −2.21 kg). The results from this BPBH trial and other recent research in this age group [19,30,45] suggest that, while e-health technologies have a role to play in treatment provision, they are not the sole solution, and some human interaction may be required. Therefore, future trials should further explore the effectiveness of different combinations of treatment modalities for weight loss among young women. Effectiveness should consider weight, behaviour and health-related outcomes, as well as reach, retention and engagement.

## Figures and Tables

**Table 1:** Description of the Be Positive Be Health*e* six-month weight loss program components.

Technology	Description ^1^
Be Positive Be Health*e* Website Participants had password-protected log-ins	Resources provide advice on weight loss, general healthy eating and physical activity, and the 10 Steps to Success. These website resources remained static over the 6 months.
	An online quiz with individualized email *feedback* in Week 1 to assess current weight, motivations, weight loss readiness, and behaviours of the 10 Steps to Success. Participants received automated *personalized* email *feedback* from their accredited practising dietitian (APD) focusing on: setting a realistic weight loss goal, their energy requirements for weight loss, and their current eating behaviours and physical activity levels compared to the 10 Steps to Success.
	Weight and behaviour change *goals* for each 10 Steps to Success were recorded in Week 1 following receipt of email feedback.
	Follow-up online quizzes (Weeks 3, 8, 12, 20) *monitored* progress towards goal achievement and APD provides automated *personalized* email *feedback* including virtual *rewards* (i.e., diamonds).
Self-monitoring app Easy Diet Diary (Xyris Software Australia) Downloaded by participants and accessible via iPhone, iPad or iPod touch (Apple Inc., Cupertino, California, USA).	Used to record weight, energy intake and energy expenditure *goals*, and to *self-monitor* weight, food intake and physical activity. Provided automated feedback on nutrient content of food items and energy expended from exercises, as well as cumulative daily totals compared to goals. Participants were recommended to record weight one/week and food intake and physical activity at least four days/week.
Email newsletters, Hypertext Markup Language (HTML) newsletters sent to participants elected email address (Campaign Monitor) Text messages (SMS) Sent to participants elected mobile number (Twilio)	Email newsletters and text messages: provided practical tips to achieve and maintain 10 Steps to Success; reminded participants to complete other program tasks (e.g., quiz, self-monitoring, and goal setting); and promoted *self-reward* for achievement of goals and *self-instruction*. During Weeks 1–12 eNewsletters were sent once/week and text messages two/week; from Week 13–26 eNewsletters were sent fortnightly and text messages one/week.
Social Media Participants joined a private Facebook group and followed a private Instagram account using their personal account Content posted using Hootsuite	Provided dynamic content about 10 Steps to Success; created a social network for women to interact with each other (*social support*) and their APD. Three posts/week made by the APD (one educational, one weekly challenge, one practical tip for overcoming barriers) plus one reminder post on weeks other tasks were to be completed (e.g., online quizzes). Both accounts moderated by the APD and questions answered.

^1^ Social cognitive theory/control theory constructs in *italics*.

**Table 2:** Baseline characteristics of young women (*n* = 57) participating in the Be Positive Be Health*e* six-month weight loss program randomized controlled trial.

Variable	Intervention (*n* = 29)	Control (*n* = 28)	All (*n* = 57)
	*n* (%)	*n* (%)	*n* (%)
Age (years) (mean ± SD)	26.3 ± 4.3	27.9 ± 5.0	27.1 ± 4.7
Country of birth			
Australia	28 (96.6)	24 (85.7)	52 (91.2)
Other	1 (3.5)	4 (14.3)	5 (8.8)
Marital status			
Never married	15 (51.7)	13 (46.4)	28 (49.1)
Married/de facto	13 (44.8)	14 (50.0)	27 (47.4)
Separated/divorced	1 (3.5)	1 (3.6)	2 (3.5)
Currently studying			
Full-time student	9 (31.0)	8 (28.6)	17 (29.8)
Part-time student	6 (20.7)	6 (21.4)	12 (21.1)
Not a student	14 (48.3)	14 (50.0)	28 (49.1)
Highest education level completed			
Higher university (e.g., Masters, PhD)	3 (10.3)	1 (3.6)	4 (7.0)
University degree	13 (44.8)	7 (25.0)	20 (25.1)
Certificate/diploma	6 (20.7)	9 (32.1)	15 (26.3)
Trade/apprenticeship	1 (3.5)	1 (3.6)	1 (3.5)
Currently studying for first degree	6 (20.7)	9 (32.1)	15 (26.3)
None	0 (0.0)	1 (3.6)	1 (1.8)
Individual income			
Lower ($0–$299/week)	9 (31.0)	4 (14.3)	13 (22.8)
Middle ($300–$999/week)	9 (31.0)	16 (57.1)	25 (43.9)
Higher ($1000 or more/week	11 (37.9)	8 (28.6)	19 (33.3)
Weight (kg)	79.8 (10.0)	79.2 (10.3)	79.5 (10.1)
Body mass index (kg/m^2^)	29.3 (2.5)	29.4 (2.5)	29.4 (2.5)
Body fat (kg)	30.2 (7.5)	30.1 (6.6)	30.2 (7.0)
Body fat (%)	37.5 (6.1)	37.9 (5.4)	37.7 (5.7)
Waist circumference (cm)	88.8 (9.0)	88.2 (8.0)	88.5 (8.5)
Blood pressure			
Systolic (mmHg)	111.9 (10.6)	112.8 (6.2)	112.3 (8.7)
Diastolic (mmHg)	73.8 (8.0)	75.4 (8.4)	74.6 (8.2)
Blood lipids			
Total cholesterol (mmol/L) ^1^	5.0 (1.0)	5.0 (0.9)	5.0 (0.9)
LDL-C (mmol/L) ^2,3^	2.8 (0.9)	2.9 (0.6)	2.8 (0.8)
HDL-C (mmol/L) ^2,4^	1.5 (0.4)	1.5 (0.4)	1.5 (0.4)
Triglycerides (mmol/L) ^1^	1.2 (0.3)	1.2 (0.6)	1.2 (0.4)
Physical activity			
MVPA minutes/week ^5,6^	243 (268)	167 (164)	208 (227)
Sitting time (minutes/day) ^7^			
Weekday	598 (266)	605 (268)	602 (264)
Weekend day	508 (227)	521 (218)	515 (221)
Average of days	567 (217)	579 (227)	573 (220)
Dietary intake			
Energy intake (kJ/day)	9106 (3483)	7840 (3828)	8484 (3680)
Alcohol (% energy/day)	0.4 (0.6)	0.5 (1.1)	0.5 (0.9)
Alcohol (grams/day)	1.5 (1.6)	1.3 (1.8)	1.4 (1.7)
Take-away (% energy/day)	5.3 (3.3)	5.7 (3.6)	5.5 (3.4)
Fruit (% energy/day)	4.9 (4.4)	4.8 (3.0)	4.9 (3.7)
Fruit (grams/day)	133.2 (71.3)	127.3 (98.2)	130.3 (84.9)
Vegetables (% energy/day)	9.6 (4.0)	9.8 (4.9)	9.7 (4.4)
Vegetables (grams/day)	427.5 (185.6)	322.0 (199.8)	375.7 (198.3)
Core foods (% energy/day)	53.6 (13.6)	56.3 (10.4)	54.9 (12.1)
Non-core foods (% energy/day)	46.3 (13.6)	43.7 (10.4)	45.0 (12.1)
Quality of life			
QLESQ ^8^ total score	46.5 (8.9)	47.1 (8.2)	46.8 (8.5)
Satisfaction with life scale	22.6 (6.2)	20.5 (6.6)	21.5 (6.5)

^1^ Total cholesterol and triglyceride analysis conducted on 56 participants, ^2^ LDL-C and HDL-C analysis conducted on 51 participants, ^3^ low-density lipoprotein cholesterol, ^4^ high-density lipoprotein cholesterol, ^5^ moderate-vigorous physical activity, ^6^ physical activity data available for 51 participants, ^7^ sitting time data available for 53 participants, ^8^ Quality of Life, Enjoyment and Satisfaction Questionnaire.

**Table 3:** Primary and secondary outcome changes in young women (*n* = 57) participating in the Be Positive Be Health*e* six-month weight loss program randomized control trial (RCT) from baseline to 6 months using intention-to-treat (ITT) analysis.

Outcome	Mean Change from Baseline to Six Months (95% CI)				Mean Difference between Groups (95% CI)	Group * Time *p*-Value	Effect Size
	Control (*n* = 28)	*p*-Value	Intervention (*n* = 29)	*p*-Value			
Weight (kg) ^1^	0.01 (−1.69, 1.70)	0.996	−1.94 (−3.59, −0.29)	**0.022**	−1.94 (−4.31, 0.42)	0.107	−0.19
Weight (kg) ^2^	0.55 (−1.28, 2.37)	0.557	−2.04 (−4.07, −0.01)	**0.049**	−2.59 (−5.32, 0.14)	0.063	−0.26
Body mass index (BMI) (kg/m^2^)	−0.01 (−0.57, 0.55)	0.970	0.69 (−1.24, −1.38)	**0.014**	−0.68 (−1.47, 1.09)	0.091	−0.27
Body fat (kg)	0.75 (−1.00, 2.49)	0.403	−2.36 (−4.27, −0.44)	**0.016**	−3.10 (−5.69, 0.52)	**0.019**	−0.44
Body fat (%)	0.27 (−1.29, 1.83)	0.733	−1.73 (−3.46, 0.003)	0.050	−2.00 (−4.33, 0.33)	0.093	−0.35
Waist circumference (cm)	−3.5 (−5.1, −1.9)	**<0.001**	−4.9 (−6.6, −3.1)	**<0.001**	−1.4 (−3.8, 1.0)	0.259	−0.16
Systolic blood pressure (mmHg)	−1.3 (−3.9, 1.3)	0.323	−4.2 (−7.1, −1.3)	**0.004**	−2.9 (−6.8, 1.0)	0.144	−0.33
Diastolic blood pressure (mmHg)	−1.2 (−3.4, 1.0)	0.297	−3.0 (−5.5, −0.9)	**0.015**	−1.9 (−5.1, 1.4)	0.267	−0.23
Total cholesterol	−0.10 (−0.46, 0.25)	0.573	−0.49 (−0.86,−0.12)	**0.009**	−0.39 (−0.90, 0.12)	0.136	−0.43
LDL-C ^3^	−0.19 (−0.61, 0.24)	0.391	−0.34 (−0.67, 0.003)	0.071	−0.15 (−0.69, 0.39)	0.581	−0.19
HDL-C ^4^	0.10 (−0.03, 0.23)	0.127	−0.13 (−0.27, 0.002)	0.054	−0.23 (−0.42, −0.05)	**0.015**	−0.58
Triglycerides	−0.18 (−0.40, 0.04)	0.114	−0.05 (−0.28, 0.18)	0.674	0.13 (−0.19, 0.45)	0.427	0.33
MVPA minutes/week ^5^	38 (−9, 165)	0.560	−20 (−141, 102)	0.749	−58 (−233, 118)	0.521	−0.25
MPA minutes/week	67 (−20, 153)	0.132	11 (−72, 94)	0.789	−55 (−175, 65)	0.366	−0.43
VPA minutes/week	−31 (−93, 30)	0.312	−32 (−91, 28)	0.302	0.0 (−86, 85)	1.000	0.00
Sitting time weekday (minutes/day)	−57 (−158, 44)	0.270	−35 (−136, 67)	0.502	22 (−121, 165)	0.764	0.08
Sitting time weekend (minutes/day)	−44 (−152, 64)	0.427	−61 (−166, 45)	0.258	−17 (−168, 134)	0.824	−0.08
Total sitting time (minutes/day)	−53 (−139, 34)	0.235	−44 (−132, 44)	0.325	9 (−115, 132)	0.892	0.04
Energy intake (kJ/day)	−106 (−1348, 1136)	0.867	−910 (−2100, 280)	0.134	−804 (−2524, 916)	0.359	−0.22
Fruit (% energy/day)	−0.73 (−2.16, 0.71)	0.323	0.75 (−0.63, 2.24)	0.388	1.47 (−0.52, 3.47)	0.148	0.40
Fruit (grams/day)	8.83 (−21.00, 38.67)	0.562	30.49 (1.94, 59.03)	**0.036**	21.65 (−19.64, 62.95)	0.304	0.26
Vegetable (% energy/day)	−1.74 (−3.55, 0.07)	0.059	2.97 (1.23, 4.71)	**<0.001**	4.71 (−2.20, 7.22)	**<0.001**	1.07
Vegetable (grams/day)	12.86 (−39.47, 65.18)	0.630	54.47 (4.46, 104.48)	**0.033**	**41.61** **(−30.77, 113.99)**	0.260	0.21
Alcohol (% energy/day)	0.03 (−0.28, 0.35)	0.842	−0.13 (−0.43, 0.17)	0.384	−0.17 (−0.60, 0.27)	0.456	−0.19
Alcohol (grams/day)	0.35 (−0.12, 0.81)	0.144	−0.34 (−0.78, 0.11)	0.135	−0.69 (−1.33, 0.04)	**0.037**	−0.41
Takeaway (% energy/day)	0.02 (−1.18, 1.23)	0.970	−1.32 (−2.48, −0.16)	**0.025**	−1.34 (−3.01, 0.33)	0.116	−0.39
% energy from non-core foods	0.89 (−4.67, 6.45)	0.754	−8.34 (−13.68, −3.00)	**0.002**	−9.23 (−16.94, 1.52)	**0.019**	−0.76
% energy from core foods	−0.89 (−6.47, 4.69)	0.754	8.50 (3.10, 13.82)	**0.002**	9.35 (1.61, 17.09)	**0.018**	0.77
QLESQ total score ^6^	2.10 (−1.27, 5.50)	0.222	3.27 (−0.39, 6.59)	0.053	1.17 (−3.57, 5.90)	0.630	0.14
Satisfaction with life scale	0.69 (−1.18, 2.57)	0.469	0.35 (−1.44, 2.14)	0.700	−0.34 (−2.94, 2.26)	0.797	−0.05

^1^ Weight including self-report, ^2^ measured weight only (*n* = 34), ^3^ low-density lipoprotein cholesterol, ^4^ high-density lipoprotein cholesterol, ^5^ moderate-vigorous physical activity, ^6^ Quality of Life, Enjoyment and Satisfaction Questionnaire; statistically-significant *p*-values (<0.05) in bold.

**Table 4:** Acceptability measures of the Be Positive Be Health*e* six-month weight loss program components.

Acceptability Measures ^1^	Website	Website Quizzes	Website: Goal Setting	Self-Monitoring App	eNewsletter	Text Messages	Social Media
Useful information about healthy eating	3.6 ± 0.8	3.5 ± 1.0	N/A ^2^	3.5 ± 0.9	3.6 ± 0.7	3.4 ± 1.0	3.8 ± 0.7
Useful information about exercise	3.4 ± 0.7	3.3 ± 1.1	N/A ^2^	3.2 ± 1.0	3.4 ± 0.7	3.2 ± 1.1	3.8 ± 0.6
Useful information about weight loss	3.4 ± 0.9	3.5 ± 0.9	N/A ^2^	2.9 ± 1.2	3.6 ± 0.7	3.4 ± 1.1	3.7 ± 0.7
Helped me attain my goals	3.0 ± 1.1	3.1 ± 1.0	3.3 ± 1.1	3.4 ± 0.9	3.1 ± 0.9	2.8 ± 0.7	3.0 ± 0.8
Motivated me	2.9 ± 1.0	3.2 ± 1.0	3.4 ± 1.0	3.6 ± 1.0	3.2 ± 0.8	2.9 ± 0.8	3.2 ± 1.0
Made me feel accountable	3.1 ± 0.9	3.4 ± 1.0	3.5 ± 1.0	3.8 ± 0.9	3.2 ± 0.8	3.0 ± 0.8	3.3 ± 1.0
Was easy to access	3.4 ± 0.9	3.8 ± 0.9	3.9 ± 0.8	3.8 ± 0.8	3.8 ± 0.8	3.6 ± 1.0	4.0 ± 0.7
Visually appealing	3.4 ± 0.8	3.6 ± 1.0	N/A ^2^	4.0 ± 0.5	3.9 ± 0.8	3.0 ± 0.9	4.0 ± 0.7
Component satisfaction	3.0 ± 1.0	3.4 ± 0.9	3.8 ± 0.7	3.8 ± 0.8	3.7 ± 0.8	3.5 ± 0.8	3.7 ± 0.8

^1^ All measures scored from 0–5, ^2^ N/A indicates not applicable (i.e., was not assessed) for this program component.

## Supplementary Materials

The following are available online at http://www.mdpi.com/2227-9032/6/2/39/s1: Table S1: Primary and secondary outcome changes in young women (*n* = 43) who completed the Be Positive Be Health*e* six-month weight loss program randomized controlled trial from baseline to six months.
