# Supplementary material for: A Targeted and Tailored eHealth Weight Loss Program for Young Women: The Be Positive Be Healthe Randomized Controlled Trial
Source: Healthcare (Basel). 2018 May 2;6(2):39. doi: 10.3390/healthcare6020039 (PMC6023329; doi:10.3390/healthcare6020039)
Supplement: Supplementary file 1 [file healthcare-06-00039-s001.pdf]

Supplementary Table 1. Primary and secondary outcome changes in young women (n=43) who completed the Be Positive Be Healthe 6-month weight loss program randomised controlled trial from baseline to 6 months.

Outcome	Mean change from baseline to 6-months (95%CI)				Mean difference between groups (95% CI)	Group x Time P-value	Effect size
	Control (n=21)	p-value	Intervention (n=22)	p-value			
Weight (kg) [1]	0.03 (-1.68, 1.74)	0.974	-1.87 (-3.54, -0.20)	0.028	-1.90 (-4.29, 0.49)	0.119	-0.20
Weight (kg) [2]	0.51 (-1.34, 2.35)	0.592	-1.83 (-3.91, 0.25)	0.084	-2.34 (-5.12, 0.45)	0.100	-0.24
BMI (kg/m^2)	-0.01 (-0.66, 0.64)	0.977	-0.69 (-1.32, -0.05)	0.034	-0.68 (-1.58, 0.23)	0.143	-0.27
Body fat (kg)	0.46 (-1.39, 2.30)	0.627	-1.75 (-3.83, 0.32)	0.098	-2.21 (-4.99, 0.57)	0.119	-0.32
Body fat (%)	0.03 (-1.6, 1.7)	0.970	-1.3 (-3.2, 0.5)	0.152	-1.4 (-3.8, 1.1)	0.273	-0.23
Waist circumference (cm)	-3.4 (-5.1, -1.8)	<0.001	-4.4 (-6.2, -2.5)	<0.001	-0.9 (-3.4, 1.5)	0.459	-0.11
Systolic blood pressure (mmHg)	-1.0 (-3.6, 1.7)	0.484	-3.8 (-6.8, -0.8)	0.013	-2.9 (-6.8, 1.1)	0.161	-0.33
Diastolic blood pressure (mmHg)	-1.2 (-3.4, 1.1)	0.300	-3.2 (-5.7, -0.6)	0.014	-2.0 (-5.4, 1.4)	0.250	-0.24
Total cholesterol	-0.1 (-0.5, 0.3)	0.648	-0.5 (-0.9, -0.1)	0.014	-0.4 (-0.9, 0.1)	0.143	-0.41
LDL-C [3]	-0.1 (-0.5, 0.3)	0.651	-0.3 (-0.7, 0.02)	0.068	-0.2 (-0.8, 0.3)	0.430	-0.29
HDL-C [4]	0.1 (-0.1, 0.2)	0.197	-0.1 (-0.3, 0.004)	0.057	-0.2 (-0.4, -0.03)	0.023	-0.44
Triglycerides	-0.2 (-0.5, 0.03)	0.088	-0.1 (-0.3, 0.2)	0.640	0.2 (-0.2, 0.5)	0.401	0.32
MVPA minutes/ week [5]	20 (-121, 161)	0.784	-39 (-175, 97)	0.575	-59 (-254, 137)	0.557	-0.24
MPA minutes/week	65 (-32, 162)	0.189	-4 (-98, 90)	0.932	-69 (-204, 66)	0.315	-0.49
VPA minutes/week	-49 (-116, 17)	0.145	-35 (-100, 30)	0.293	15 (-78, 107)	0.759	0.10
Sitting time weekday (mins/day)	-78 (-178, 23)	0.129	-10 (-111, 92)	0.850	68 (-75, 211)	0.351	0.28
Sitting time weekend (mins/day)	-65 (-176, 46)	0.250	-64 (-172, 44)	0.246	1 (-154, 156)	0.991	0.00
Total sitting time (mins/day)	-71 (-158, 16)	0.109	-26 (-114, 62)	0.559	45 (-79, 168)	0.476	0.22
Energy intake (kJ/day)	-191 (-1484, 1102)	0.772	-982 (-2215, 251)	0.119	-791 (-2578, 996)	0.386	-0.21
Fruit (% energy/day)	-0.60 (-2.10, 0.90)	0.433	1.14 (-0.29, 2.57)	0.119	1.74 (-0.33, 3.81)	0.100	0.45
Fruit (grams/day)	10.08 (-20.40, 40.55)	0.517	35.55 (6.49, 64.61)	0.016	25.48 (-16.64, 67.59)	0.236	0.31

Vegetable (% energy/day)	-1.55 (-3.46, 0.36)	0.111	2.91 (1.09, 4.73)	0.002	4.46 (-1.82, 7.10)	0.001	0.92
Vegetable grams/day	15.43 (-38.33, 69.18)	0.574	57.05 (5.80, 108.31)	0.029	41.63 (-32.64, 115.90)	0.272	0.19
Alcohol (% energy/day)	5.55e-17 (-0.32, 0.32)	1.000	-0.14 (-0.44, 0.17)	0.383	-0.14 (-0.58, 0.31)	0.547	-0.14
Alcohol (grams/day)	0.33 (-0.15, 0.80)	0.180	-0.36 (-0.82, 0.09)	0.119	-0.69 (-1.34, 0.03)	0.041	-0.38
Takeaway (% energy/day)	0.30 (-0.93, 1.53)	0.634	-1.36 (-2.54, -0.19)	0.023	-1.66 (-3.37, 0.04)	0.056	-0.48
% energy from non-core foods	1.0 (-4.93, 6.93)	0.741	-7.36 (-13.01, -1.71)	0.011	-8.36 (-16.55, 0.18)	0.045	-0.67
% energy from core foods	-1.0 (-6.95, 4.95)	0.742	7.50 (1.83, 13.17)	0.010	8.50 (-0.28, 16.72)	0.043	0.68
QLESQ total score [6]	1.8 (-1.7, 5.3)	0.312	2.8 (-0.6, 6.2)	0.107	1.0 (-3.9, 5.9)	0.687	0.12
Satisfaction with life scale	0.6 (-1.3, 2.5)	0.530	0.2 (-1.6, 2.0)	0.844	-0.4 (-3.0, 2.2)	0.751	-0.27

[1] Weight including self-report, [2] Measured weight only, [3] Low density lipoprotein cholesterol, [4] High density lipoprotein cholesterol, [5] Moderate-vigorous physical activity, [6] Quality of Life, Enjoyment & Satisfaction Questionnaire
